# GHWR, a multi-method global heatwave and warm-spell record and toolbox

**DOI:** 10.1038/sdata.2018.206

**Published:** 2018-10-30

**Authors:** Ehsan Raei, Mohammad Reza Nikoo, Amir AghaKouchak, Omid Mazdiyasni, Mojtaba Sadegh

**Affiliations:** 1 Shiraz University, Shiraz, Iran; 2 University of California, Irvine, CA, USA; 3 Boise State University, Boise, ID, USA

**Keywords:** Atmospheric dynamics, Environmental chemistry

## Abstract

Heatwaves are extended periods of unusually high temperatures with significant societal and environmental impacts. Despite their significance, there is not a generalized definition for heatwaves. In this paper, we introduce a multi-method global heatwave and warm-spell data record and analysis toolbox (named GHWR). In addition to a comprehensive long-term global data record of heatwaves, GHWR allows processing and extracting heatwave records for any location efficiently. We use traditional constant temperature threshold methods, as well as spatially and temporally localized threshold approaches to identify heatwaves. GHWR includes binary (0/1) occurrence records of heatwaves/warm-spells, and annual summary files with detailed information on their frequency, duration, magnitude and amplitude. GHWR also introduces the standardized heat index (SHI) as a generalized statistical metric to identify heatwave/warm-spells. SHI has direct association with the probability distribution function of long-term daily temperatures for any given calendar day and spatial grid. Finally, GHWR offers a unique opportunity for users to select the type of heatwave/warm-spell information from a plethora of methods based on their needs and applications.

## Background & Summary

Heatwaves are generally defined as prolonged periods of high temperature and excess heat^1^ with significant impacts on humans and natural ecosystems. In the recent decades, heatwaves have become one of the deadliest natural hazards in some regions^2,3^, and are recognized as *“silent killers”*^4^. Heatwaves leave marked societal impacts ranging from damaging infrastructure, stressing emergency response units and hospitals, and overburdening the electricity grids, to paralyzing the railroad and air transportation systems^2,5–7^. The extent of such impacts depends largely on the societal preparedness to respond to the extreme events^3,8^. Other negative impacts of heatwaves and warm-spells include reducing work capacity and labor productivity^9^, crop yield^9,10^, and livestock productivity^2^.

A comprehensive analysis of heatwaves should consider four key characteristics of such events, namely: 1. intensity, 2. duration, 3. frequency, and 4. spatial extent^1,2,11–13^. In this study, we create a comprehensive multi-method global heatwave and warm-spell record and toolbox, GHWR, based on different methodologies in the literature. Here, a warm-spell corresponds to unusually warm temperatures even if they are not extremely high (e.g., a 5-day event in winter with an average temperature of + 5 °C in a region where the expected average temperature based on long-term climatology is −5 °C). GHWR can be used to analyze changes in statistics of heatwaves and warm-spells around the world, and to quantify their impacts on different sectors and target groups.

Heatwave and warm-spell identification methodologies in this data set include five general categories: 1. constant temperature threshold approach, 2. location and calendar day specific temperature threshold (PDF-derived threshold approach), 3. location specific threshold based on upper tail of summer temperature distribution (summer-derived threshold approach), 4. excess heat factor (EHF) approach, and 5. the herein introduced standardized heat index (SHI) method. Heatwaves are complex in nature and cannot be simply compared across regions, natural systems, and target sectors^14^. The idea behind SHI is to provide standardized and spatially consistent heatwave and warm-spell information. SHI and EHF methods employ average daily temperature, while other approaches are based on daily max, min or average temperature.

In this data record, we cover a wide range of temperature thresholds to address different potential applications. The threshold approaches (items 1–3), consider 2, 3, 4, 5, 6, 7 and 10 consecutive days as required temporal persistence for heatwave and warm-spells. The GHWR data set is generated using the CPC global air temperature data provided by the NOAA/OAR/ESRL PSD, Boulder, Colorado, USA (http://www.esrl.noaa.gov/psd/). Gridded air temperature at the surface is provided for the entire globe with a spatial resolution of 0.5° and a daily temporal resolution for 1979 to present. GHWR incorporates all the required characteristics of heatwaves and warm-spells (intensity-duration-frequency-spatial extent) in one easily accessible depository, and can be used in different studies ranging from human mortality and morbidity, impacts on the energy and agriculture sectors, and also spatiotemporal statistical analysis of heatwaves and warm-spells. It is noteworthy that GHWT (toolbox) relaxes the dependency of GHWR (data set) on the source CPC temperature data, and can perform a comprehensive heatwave/warm-spell analysis on any gridded daily temperature source data for any location.

Existing heatwave datasets mainly focus on fixed-threshold definitions emanated from community efforts such as the Expert Team on Climate Change Detection and Indices (ETCCDI)^15,16^. Hadley Extremes database (HadEX)^17^ (http://www.metoffice.gov.uk/hadobs/hadex2/), for example, provides coarse resolution ETCCDI indices on a 2.5°×3.75° grid from 1901 to 2010. Hadley Centre/Global Historical Climatology Network (HadGHCND) extremes database (GHCNDEX) compute such indices based on ground-based station data^18,19^. However, over a relatively short period of time, significant strides have been made in characterizing heatwaves and understanding their impacts^1^, some of which call for or propose localized and sector-specific thresholds for identifying heatwaves^1,2,14,20–22^. GHWR leverages the recent contributions in the literature, as well as the ETCCDI indices, to create a multi-method global heatwave and warm-spell record based on gridded 0.5 °×0.5 ° temperature data from 1979 to 2017. In more detail, GHWR leverages a plethora of different methods (discussed in Methods) with various settings (see Table 1) to enable future users to compare different heatwave definitions and select the most proper method for the target study.

## Methods

A heatwave is generally defined as temperatures rising above a certain threshold (either percentile-based or fixed value) for a number of consecutive days. Despite the strides made in the field, there is no universal consensus for defining heatwaves^4^. A plethora of definitions are available in the literature^23^ each focusing on a particular application or target group (e.g. human morbidity and mortality, infrastructure and transportation systems, wildfire, and agriculture sector)^14,24^. However, all definitions of heatwaves involve at least one form of temperature (daily max, min, or average) and require a predefined threshold to be exceeded for a number of consecutive days^1^. The specification of threshold is of highest significance, and is dependent on the application or the target group of study (e.g., plants, humans, energy sector).

Even a slight change in heatwave definition might lead to considerable effects on the estimated impacts^25^. Some animals are sensitive to a specific range of temperatures, and some agricultural products show a direct response to a particular temperature threshold^26^. For example, flying foxes (native to Australia) may die from dehydration at air temperatures of above 40 °C, especially when relative humidity is low^2^. Human’s core temperature is 37 °C, on average, and a rise to 38 °C for several hours may lead to heat exhaustion and may impair physical and mental capacities^27,28^. The temperature threshold for heatwaves may also be selected based on the climatology of a region^3^, such as the 90^th^ percentile of the local daily temperature distribution, since humans and the natural ecosystem acclimate to the local climate^25^. Moreover, temperature thresholds could/should differ for individual calendar days. For example, in a region with strong seasonality, identifying warm-spells in winter requires a much lower temperature threshold than that in summer^1^.

In the following, we provide a comprehensive, but non-exhaustive, list of definitions from the literature, and introduce a new standardized and probabilistic definition. The below description focuses mainly on the heatwave definitions used in GHWR (also see Table 1). The user should note that GHWR provides a plethora of different methods to detect heatwaves and warm-spells, but not all methods are necessarily appropriate for all regions and or applications. The user should carefully select the proper method based on the region and application of interest. However, a unique feature of GHWR is indeed enabling users to contrast and compare different approaches.

### Constant temperature threshold approach

This approach defines a heatwave as an event during which temperatures exceed a constant threshold for an extended period of time^25,29–35^. In GHWR, the extended period of time is considered 2, 3, 4, 5, 6, 7 and 10 consecutive days during which daily max, min, or average temperature surpasses a wide range of thresholds (35, 36, 37, 38, 39, 40, 41, 42, 43, 44, 45 °C for daily maximum temperature, TMAX; 25, 26, 25, 28, 29, 30, 31, 32, 33, 34, 35 °C for daily minimum temperature, TMIN; and 30, 31, 32, 33, 34, 35, 36, 37, 38, 39, 40 °C for daily average temperature, TMEAN). In other words, GHWR provides different global heatwave records using different combinations of temperature threshold and duration for daily max, min and average temperature observations (e.g., all 5-day heatwave events with daily maximum temperature greater than 35 °C).

### PDF-derived temperature threshold

Temperature thresholds for heatwaves, in this approach, are assigned according to the local climatology. For example, events during which temperature observations exceed a localized threshold (e.g., 90^th^ percentile) for several consecutive days are defined as heatwaves^36–38^. GHWR provides heatwave and warm-spell information based on different PDF-derived thresholds using daily max, min and average temperatures.

### Average temperature+5degC

This approach considers a spatially localized temperature threshold to delineate heatwave days from non-heatwave ones^1,39^. This method only considers daily average temperatures with a temperature threshold that is estimated as the mean of long-term daily average temperature observations + 5 °C. Note that this is an outdated approach that neglects the relative climatological features of the region, and might not be applicable everywhere. As a matter of fact, in some regions temperature might not even get 5 °C above average climatological temperature^40^. However, we have included it for cross comparison of different methods.

### Upper tail percentile

This method localizes temperature threshold both spatially and temporally. In more detail, temperature thresholds for each calendar day and each grid are specified as an upper tail threshold (75^th^, 80^th^, 85^th^, 90^th^, 95^th^ percentiles) of the probability distribution function (PDF) constructed from long-term daily temperature record over a window of 15 (21) days centered around the calendar day of interest^11,12,41–43^. Temporal localization in this approach enables detection of warm-spells (e.g., unusually warm winter) as well as heatwaves.

### Summer-derived threshold approach

This method spatially localizes temperature threshold for heatwaves, and assigns an upper tail threshold (75^th^, 80^th^, 85^th^, 90^th^, 95^th^ percentiles) of the PDF constructed from long-term daily summer (warm season) temperatures in each grid^3,44^. This approach is conceptually similar to the PDF-derived threshold but only uses data from summer (warm season), and does not consider temporally localized thresholds. This definition is more appropriate for studies that focus on extreme heat and their impacts on mortality, crop yield, etc.^3^. GHWR provides heatwave information based on this definition using daily max, min and average observed temperatures, and different duration (2, 3, 4, 5, 6, 7 and 10 consecutive days).

### Excess heat factor (EHF)

Excess heat factor (EHF) combines measures of *“excess heat”* and *“heat stress”* to develop an index that can effectively describe the impacts of heatwaves on human mortality and morbidity^14,21,41^. Excess heat characterizes localized long-term temperature anomaly compared to climatology of the region (grid), whereas heat stress represents short-term temperature anomalies to address thermal acclimation^22^. EHF is constructed based on a running three-day-averaged TMEAN (daily average temperature), as vulnerable population show highest sensitivity to heatwaves after three days of exposure^41^. EHF is specifically designed to capture heatwave severity^22^.

High day-time heat accumulates if night-time temperatures are high as well. Average of daily maximum and minimum temperature over a three day running period can then represent the accumulated excess heat, when compared to a long-term climatological baseline (reference)^41^. The long-term climate reference is set to 95^th^ percentile of the PDF of daily average temperature (hereafter, *T*_95_). Positive and consecutive anomaly of a three-day-averaged mean temperature from this reference value is indicative of a heatwave event. Excess heat index is formulated as,

(1)EHIsig=(Ti+Ti+1+Ti+2)/3-T95,

in which, *T*_*i*_ denotes mean daily temperature at day *i*. Heat stress, reflecting short-term acclimation, is then defined as,

(2)EHIaccl=(Ti+Ti+1+Ti+2)/3-(Ti-1+Ti-2+⋯+Ti-30)/30.

Finally, EHF aggregates the impacts of EHI_sig_ and EHI_accl_ to develop a comparative index representing intensity, load, duration and spatial distribution of a heatwave event^41^,

(3)EHF=EHIsig×max(EHIaccl,1).

In GHWR, we consider EHF≥0°C^2^ and EHF≥1°C^2^ as thresholds for heatwave.

### Standardized heat index (SHI)

We introduce standardized heat index (SHI) as a probabilistic and generalized index to describe heatwaves according to their probability of occurrence in long-term records. SHI is inspired by the standardized precipitation index (SPI)^45^ and is spatially and temporally localized. For each grid (region), a temperature PDF is constructed for a window of 15 (and 21) days centered around the calendar day of interest, and the empirical probability for each temperature level is calculated using the Gringorten plotting position^46^

(4)p(Ti)=ri-0.44n+0.12,

in which *T*_*i*_ is the *i*^th^ observed mean daily temperature in the constructed PDF, and *r*_*i*_ is the rank of *T*_*i*_ from the smallest value^47^. SHI for temperature level $\hat{T}$ is then estimated as

(5)SHI(Tˆ)=Φ−1(p(Tˆ)),

where Φ signifies the standard normal distribution. This approach does not require the data to follow normal distribution, and the last step is only used to describe all heatwave information in a standardized scale (e.g., standard normal distribution).

In order to address the persistence of excess heat for an extended period of time, and similar to EHF, we consider three-day-averaged mean daily temperature $\left(\hat{T}=(T_{i}+T_{i-1}+T_{i-2})/3\right)$ to develop the SHI index (Eq. 5). We adopt a threshold of SHI>=1 for identifying heatwaves, which is associated with three-day-averaged mean daily temperature being greater than the ∼84^th^ percentile of the long-term temperature PDF (Φ(1)=0.8416). A threshold of 1 is selected herein to be synergistic with the SPI index (see National Drought Monitor for definition of dry and wet years according to SPI). The user can select other thresholds such as 1.28, 1.5, or 2 which coincide with 90^th^, 93.3^th^ and 97.7^th^ percentiles, respectively. Given the statistical basis of this index, SHI can be used to identify both coldwaves (cold-spells) and heatwaves (warm-spells), by tuning the threshold. The user can select thresholds that are more appropriate for their own studies.

### Code Availability

The GHWR source codes are developed in MATLAB and are available to public along with the GHWR data set (under “GHWR_Codes” folder). The source codes are developed based on the CPC gridded global daily temperature data set, but the user can readily execute these codes to generate heatwave record based on any gridded daily source data, and selection of heatwave method and its parameters. The user can alternatively use the global heatwave and warm-spell toolbox, GHWT, as explained in the next section.

### Global heatwave and warm-spell toolbox, GHWT

**Figure 1:**
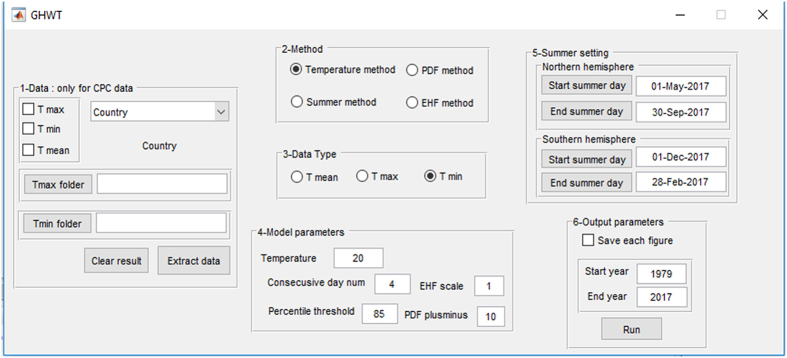
Graphical user interface (GUI) of the global heatwave and warm-spell toolbox, GHWT.

GHWT is a user-friendly and flexible toolbox developed in MATLAB to construct long-term heatwave and warm-spell record with any gridded global daily temperature data source for any country/continent around the globe. The user can select to perform computations using either the graphical user interface (GUI; execute “GHWT.m” in MATLAB) or the “run script” (modify and execute “GHWT_Script_run.m” in MATLAB). Fig. 1 displays the GHWT toolbox GUI. First step is to select temperature data directory and the country (or continent) of interest. This first step is designed for the CPC temperature data source. If the user opts to employ any other source for temperature data, (s)he should first modify the “config.txt” file and execute “extract_data.m” under the “extract other database format” subforlder to pre-process the source data. Subsequent steps can all be performed in the GUI. Next is to select the heatwave/warm-spell identification method, and selection of data type (max, min, or average daily temperature). Then, the user should define the method-specific parameters of the analysis (such as the number of consecutive days for the temperature to be above a certain threshold). If the user selects the “Summer method”, (s)he needs to define the start and end date of summer. The user subsequently selects the start and end year of analysis and execute the program. The GHWT toolbox plots several figures displaying the number of heatwave days and events, first and last heatwave day of year, length of longest heatwave, and magnitude and amplitude of heatwaves for each year. These information will also be saved under “GHWT_results.mat” under the “result” folder.

GHWT is publicly available at https://github.com/mojtabasadegh/Global_Heatwave_and_Warm_Spell_Toolbox under Attribution-ShareAlike 3.0 license (https://creativecommons.org/licenses/by-sa/3.0/). Country borders shapefiles used in this toolbox were obtained from http://thematicmapping.org/.

### Data Records

The global heatwave and warm-spell record (GHWR) is freely available to public at (Data Citation 1).

The GHWR record provides information on heatwaves including intensity, duration, frequency, and spatial extent. The gridded global heatwave record is provided as binary (0/1) and annual summary for each heatwave definition in “netcdf” format. The binary form will categorize each day as 1 if identified as heatwave/warm-spell, and 0 otherwise. The annual summary files for each definition provides detailed information about heatwave/warm-spell including: a. number of days, b. number of events, c. first heatwave occurrence day of year, d. last heatwave occurrence day of year, e. length of longest event, f. length of shortest event, g. magnitude (average of the mean heatwave/warm-spell temperatures of all events), h. amplitude (peak daily temperature in the hottest heatwave as defined by the heatwave with highest magnitude). It is also important to mention that for SHI and EHF methods, we also provide time series of EHF and SHI record for each grid over 1979–2017. Moreover, temporally and spatially localized heatwave/warm-spell thresholds for each method is included in GHWR for future and further analysis. A tabulated description of heatwave/warm-spell definitions that are used in the GHWR data set is provided in Table 1.

It is noteworthy that all files are compressed to “7z” format to reduce size, and proper MATLAB and Python codes are provided to automatically decompress the selected files to “netcdf” format. The GHWR data set is provided in three general folders namely: Record, Summary, and Threshold; which in turn are divided into two parts (e.g. Record1 and Record2) for facilitating upload to data repository. Each of these general folders then include heatwave information from all methods and possible combinations of parameters as presented in Table 1. A “Data Directory” (“pptx” format) provides guidance to find any specific file. A “readme.txt” file is also available along with the data set that explains the decompressing process.

## Technical Validation

We analyze three historical heatwave events from around the globe that led to high societal impacts using the GHWR data set. We provide evidence that the different methodologies employed to create the data set not only capture the intensity and duration of these events, but also provide important insights about the spatial extent of these events. Moreover, GHWR’s length of record (1979-2017) enables users to analyze frequency of a heatwave or warm-spell event and, placing it in a climatological perspective. GHWR also provides temporally and spatially localized heatwave/warm-spell temperature thresholds for different methods, which can in turn be used for identification of future heatwave events given temperature predictions.

### Chicago heatwave (July 1995)

The heatwave of July 1995 that affected most of the Midwest US, were unprecedented in many senses. Extreme day- and night-time temperatures persisted over an extended 48-hour period, was exacerbated by high relative humidity, and left remarkable societal impacts in Chicago, IL^48^. Due to the excessive heat and humidity, hospital admission increased 35% for the elderly (65 years and older)^24^. Multiple studies^49,50^ attributed at least 600 excess deaths to the extreme heat during and right after this event. It is noteworthy that while extreme day-time apparent temperature (an index of the combined effect of temperature and humidity on humans) in the Chicago heatwave is not particularly exceptional, the persistence of high night-time apparent temperature was unprecedented in the modern history (with probability of occurrence of less than 0.1%,^48^). Night-time lower temperatures are necessary to relieve the day-time excess heat, absence of which significantly exacerbate the impacts of heatwaves^2^.

**Figure 2:**
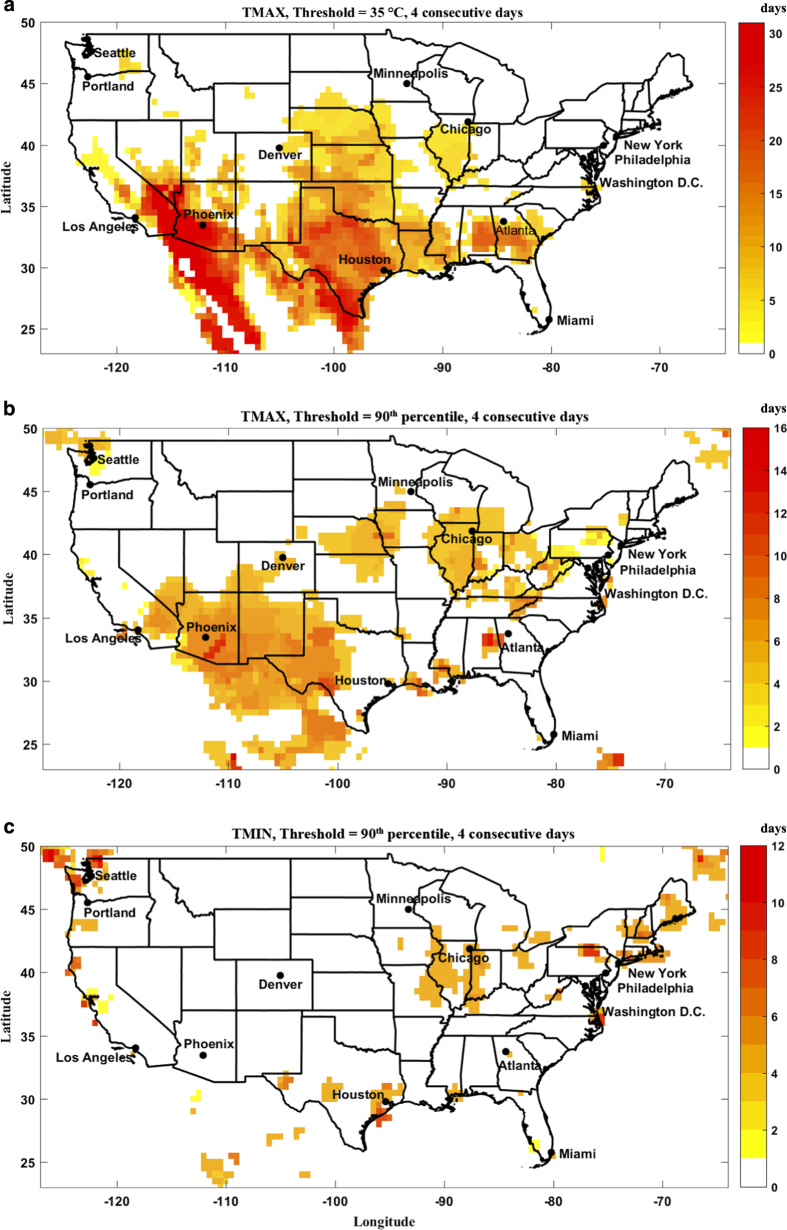
Frequency of heatwave days in the month of July, 1995 using different heatwave definitions. (**a**) Daily maximum temperature above 35 °C, (**b**) Daily maximum temperature above 90^th^ percentile of the long-term TMAX climatology, and (**c**) Daily minimum temperature above 90^th^ percentile of the long-term TMIN climatology, for at least four consecutive days.

Figure 2 shows the frequency of heatwave days in July of 1995 in the US (with a focus on Chicago, IL), based on different definitions/methods. Figure 2a displays frequency of heatwave days defined based on the maximum daily temperatures above 35 °C with a duration of at least four consecutive days. While Midwest US, and specifically Chicago, experienced around 5 heatwave days in July of 1995 according to this definition, heatwave days were much more frequent in South and Southwest US. Indeed, the entire month of July, 1995 (31 days) is categorized as heatwave days for some regions in the west of Arizona and southeast of California in this category. This highlights the need to define a local heatwave threshold for each region. Figure 2b displays frequency of heatwave days in July, 1995 for the entire US with a temperature threshold of 90^th^ percentile of the long-term TMAX distribution for each grid and each calendar day, with a temporal persistence of at least four days. It is noticeable that the geographical extent of the Midwest US heatwave expands to north of Chicago in this definition (compare to Fig. 2a). Moreover, frequency of heatwave days in the south and southeast US also significantly decreases in this definition. However, still some parts of Arizona show more frequent heatwave days compared to the Midwest US where the Chicago heatwave had left more significant impacts. This is due to the persistence of high day- and night-time temperature in the Midwest US. Figure 2c shows frequency of heatwave days with minimum (night-time) temperature above 90^th^ percentile of the long-term TMIN distribution for at least four successive days. Indeed, persistence of high day- (Fig. 2b) and night-time (Fig. 2c) temperatures explain the high impact of this event. Figure 2 clearly demonstrates the advantages of defining local thresholds for heatwaves compared to that of the constant temperature threshold approach^1^.

Note that the choice of heatwave duration, as well as other heatwave parameters, is merely to demonstrate different features of GHWR. The user can select any heatwave setting that fits the target study from hundreds of combinations available in GHWR.

### European heatwave (summer 2003)

The mega-heatwave of July and August, 2003 in Europe was the hottest period that western Europe has experienced since at least 1500 AD^51^. Driven by the anticyclonic (high atmospheric pressure) conditions and unusual soil moisture deficit^52^, the 2003 European heatwave claimed more than 70,000 lives, hitting France the hardest^53,54^. Similar to other deadly heatwaves^55^, night-time temperature during the European 2003 heatwave were significantly higher than normal^56^. Recent studies have provided considerable evidence that the rate of increase in night-time temperature is even higher than its day-time counterpart, which exacerbates the negative societal and environmental impacts of such events^2^.

**Figure 3:**
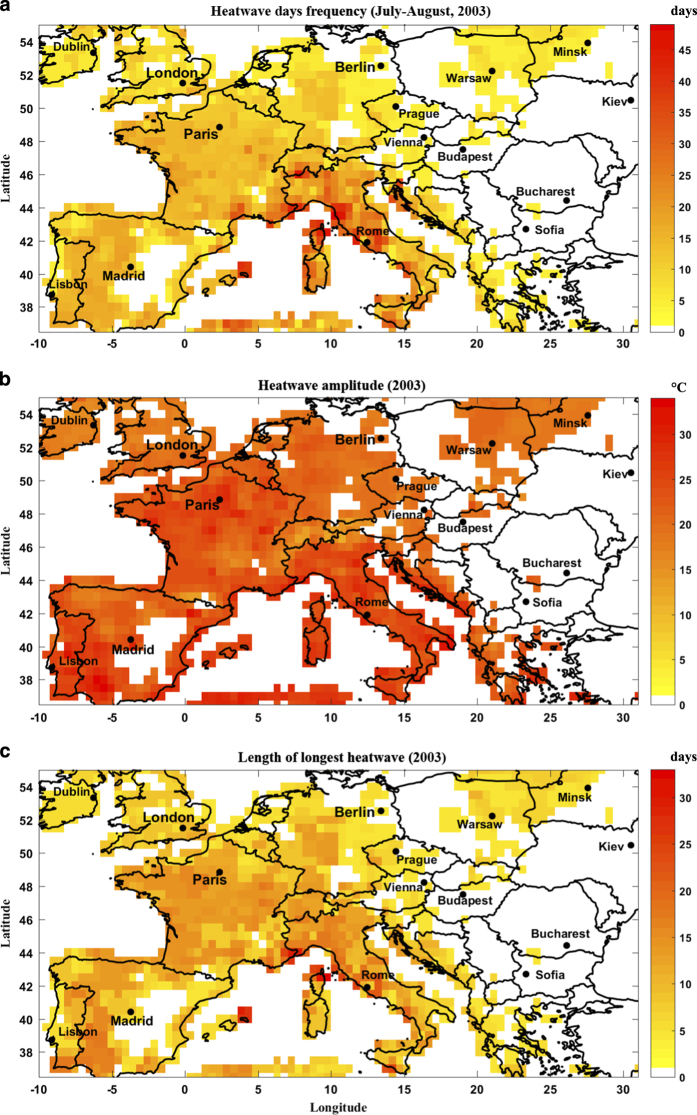
The 2003 European heatwave characteristics. (**a**) Frequency of heatwave days in July-August, 2003, (**b**) Heatwave amplitude (based on TMIN) in 2003, and (**c**) Length of the longest heatwave in 2003. Heatwave is defined as persistence of daily minimum temperature above 90^th^ percentile of TMIN climatology for at least four consecutive days.

Figure 3a displays the frequency of heatwave days over July and August, 2003 in western Europe, as defined by minimum daily temperature exceeding 90^th^ percentile of long-term distribution of TMIN for each day and each grid for at least four consecutive days. Some locations, interestingly, endure around 45 heatwave days in a two months period. Following a similar heatwave definition, Fig. 3b displays the heatwave amplitude for daily minimum temperature (as defined by the peak daily TMIN in the hottest heatwave), which exceeds a night-time temperature of 30 °C in an extended region around Paris, France. Finally, Fig. 3c shows length of the longest heatwave event in 2003. Similarly, heatwaves are defined as those with daily minimum temperature exceeding 90^th^ percentile of TMIN climatology for at least four days. Most locations in Europe experience around 10 consecutive heatwave days in 2003, which extends to 30 + days in some regions. The spatiotemporal extent of this heatwave was indeed unprecedented, only to be surpassed by the Russian 2010 heatwave.

### Russian heatwave (summer 2010)

The Russian heatwave in summer of 2010 was exceptional in many senses. It exceeded the amplitude and spatial extent of the European 2003 heatwave^12^, and persisted for over one month^57^. Indeed, July 2010 was by far the warmest on record in western Russia^58^. Similar to the European heatwave of 2003, the Russian 2010 heatwave was driven by a blocking anticyclone and exacerbated by the soil moisture-temperature feedback^59^. The soil moisture deficit in western Russia in 2010 alone increased the odds of a severe heatwave sixfold^59^. This event broke several records, and daily maximum temperatures persisted around the record levels for an extended period of time^60^. This mega heatwave claimed some 56,000 lives^57^, left negative impacts on the agriculture^1^, and contributed to 500 wildfires^1^, which in turn provoked air pollution^52^.

**Figure 4:**
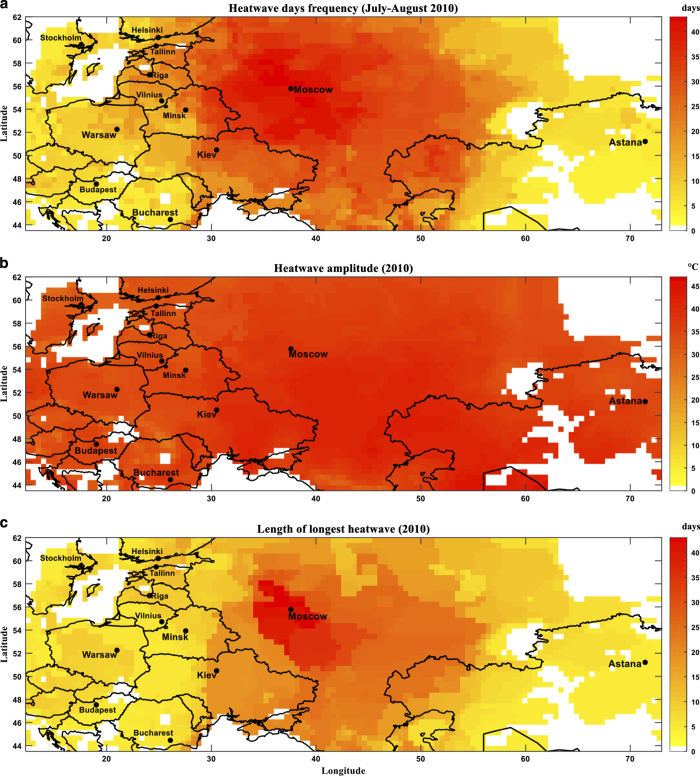
The 2010 Russian heatwave characteristics. (**a**) Frequency of heatwave days in July-August, 2010, (**b**) Heatwave amplitude (based on TMAX) in 2010, and (**c**) Length of longest heatwave in 2010. Heatwave is defined as persistence of daily maximum temperature above 90^th^ percentile of TMAX climatology for at least four consecutive days.

Figure 4a displays frequency of heatwave days in western Russia in July and August, 2010. Heatwave is defined by daily maximum temperature exceeding 90^th^ percentile of the long-term TMAX climatology for at least four consecutive days for each location and calendar day. A large region surrounding Moscow, Russia, experienced 40 + heatwave days in two months. Figure 4b shows heatwave amplitude for daily maximum temperature soaring 45 + °C for most of western Russia. Finally, length of the longest heatwave event is portrayed in Fig. 4c, showing a continuous 40 + heatwave days in a vast area around Moscow. Temporal extension of heatwaves exacerbates the heatwave impacts by restricting relief periods needed for the human and environmental systems to recover.

**Figure 5:**
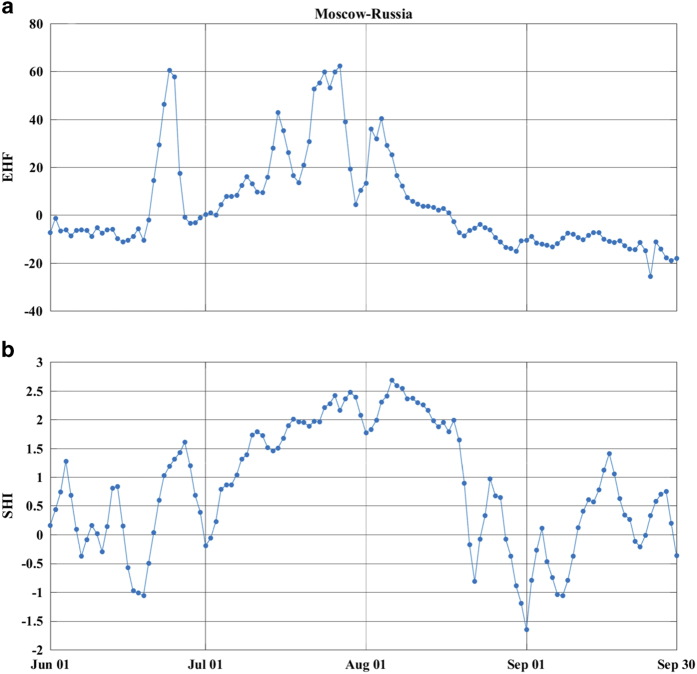
Heatwave intensity for Moscow, Russia, during June-September 2010. (**a**) Time series of excess heat factor (EHF), and (**b**) Time series of standardized heat index (SHI).

Figure 5 displays daily time series of EHF (a) and SHI (b) values for Moscow, Russia, during June-September, 2010. Both approaches capture the intensity and temporal extension of the 2010 Russian heatwave over Moscow, Russia. However, they show different behavior in late July and mid September. In late July as average daily temperature drops from ∼32 °C to ∼25 °C, EHF drastically reduces from ∼60 °C^2^ to ∼4°C^2^, whereas SHI shows a more moderate drop from ∼2.5 (∼99^th^ percentile) to ∼1.75 (∼96^th^ percentile). The different response of these two metrics is rooted in their definitions, as EHF considers acclimation to recent weather conditions (mean daily temperature of the previous 30 days) whereas SHI does not include such an effect. SHI is closer in definition to that of the traditional heatwave literature. SHI and EHF also show a different behavior in mid September, when SHI shows a heatwave/warm-spell event whereas EHF does not. Indeed, in mid September average daily temperature rises to ∼17 °C which equates to the ∼92^th^ percentile of the TMEAN climatology (15 day window centered at September 15). This shows that September 15, 2010 is unusually hot for its historical record, but since EHF is only focused on human mortality and morbidity, it would not recognize this event as warm-spell. A deeper discussion is required to recognize the similarities and differences between SHI and EHF, which is beyond the scope of this paper. It is noteworthy that SHI is a probabilistic measure with clear association with the temperature distribution, and can readily be interpreted as different percentiles of the long-term mean daily temperature PDF. Moreover, SHI can also identify cold-spells as they fall in the lower tail of the long-term TMEAN PDF.

### Global heatwave/warm-spell maps

**Figure 6:**
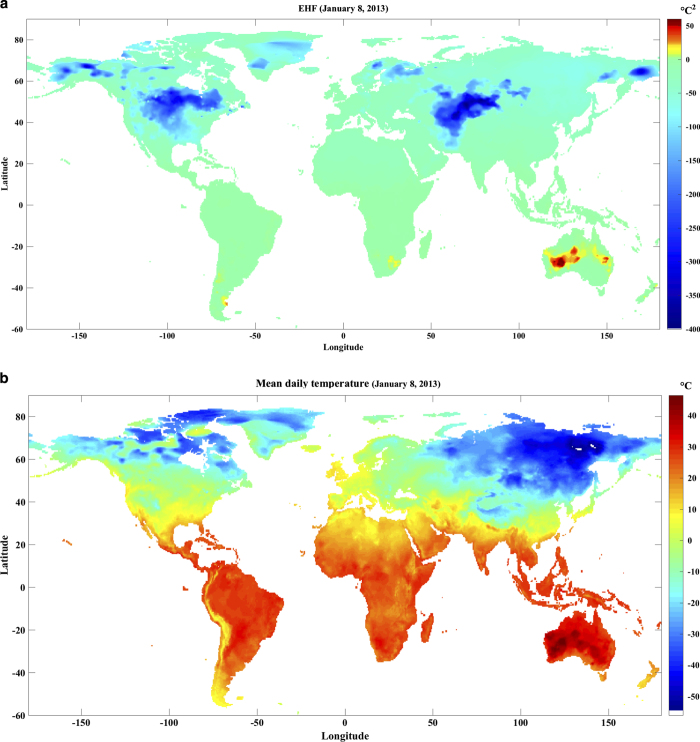
Global temperature and heatwave situation on January 8, 2013. (**a**) Excess heat factor (EHF), and (**b**) Mean daily temperature.

Figures 6a displays the EHF record for January 8, 2013 over the entire globe. January 2013 coincides with the “angry summer” in Australia, and was the hottest months on record in Australia. “Angry summer” broke the all-time TMAX record for several dozen stations across Australia^61^. EHF correctly captures the severity of the “angry summer” heatwave on January 8, 2013 for western and central Australia (EHF reaching 50 °C^2^). The mean daily temperatures soar above 45 °C on this day (Fig. 6b) for many parts of Australia, specially in the western and central regions.

**Figure 7:**
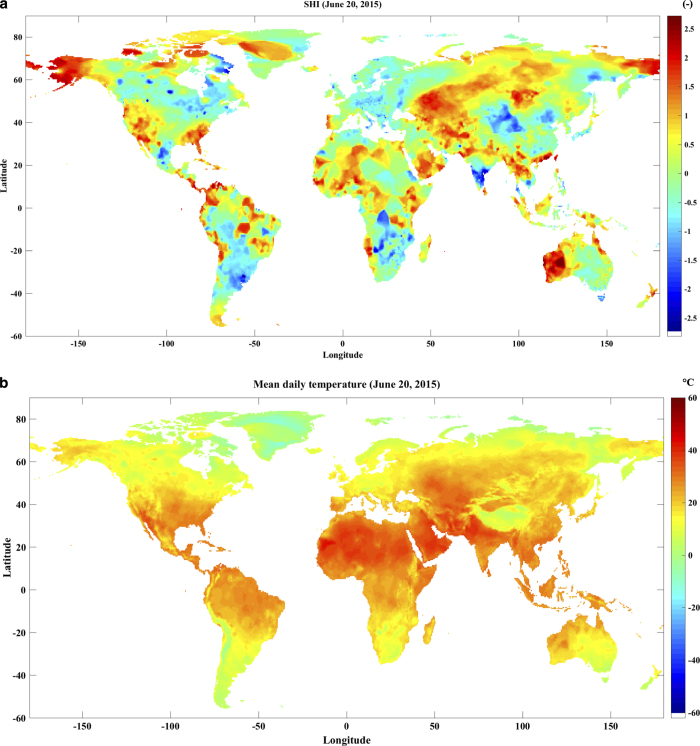
Global temperature and heatwave situation on June 20, 2015. (**a**) Standardized heat index (SHI), and (**b**) Mean daily temperature.

Figure 7a shows SHI record for June 20, 2015 over the entire globe. Karachi, Pakistan, experienced an extreme heatwave in June, 2015, which along with power outage and water distribution system failure, imposed a death toll of 700 + people^62^. This figure captures the Karachi’s heatwave with SHI>2 (daily mean temperature above 97^th^ percentile of TMEAN climatology). Figure 7a also shows heatwaves over many regions in the northern hemisphere and warm-spells in the southern hemisphere.

## Usage Notes

In this paper, we introduce a multi-method global heatwave and warm-spell record, GHWR. We leverage different definitions of heatwaves from the literature, and introduce a probabilistic index namely standardized heatwave index (SHI), that provides spatially and temporally consistent information on heatwaves and warm-spells in a standardized scale. This data set employs the CPC global air temperature data at the surface level with a spatial resolution of 0.5°, and identifies heatwave and warm-spells using different definitions. The GHWR data provides heatwaves and warm-spells information based on daily max, min and average temperature levels.

One important feature of GHWR is its focus on warm-spells alongside heatwaves. Heatwaves with direct and immediate human impacts have been widely analyzed in the literature^1^, whereas warm-spells are yet to be more rigorously studied. Warm-spells also deeply impact human lives and livelihood. For example, warm-spells have induced earlier spring snow melt, which in turn increase western US forest wildfire activity^63^. Another prominent contribution of the GHWR data set is enabling spatial analysis of heatwaves at the global scale. Note that GHWR, as well as any other global product, often does not account for the urban heat islands (UHIs), unless the UHI effect is reflected in the input data. Inner cities might be 1 to 3 °C hotter than the open areas, a phenomenon that is referred to as urban heat island^64^. This is due to a combination of *‘increased heat storage and reduced water retention capacities of the land surface’*^65^. UHIs’ major influence is on increasing the night-time temperature, which along with large exposure of vulnerable groups enhance the risks of heat waves^2^.

Finally, governments and the public should take a more proactive approach to reduce the impacts of heatwaves^66^. The sustained 2013 England heatwave is a good example of an extreme event for which preparation and proper response reduced mortality rate to much less than expected^67^. Early warning systems^68^ and science-informed strategies to help the vulnerable population^50,69^ are essential to reduce the impacts of heatwaves. However, improving our understanding of heatwave features, developing warning systems and adaption strategies all rely on data. We hope that GHWR facilitates research and data analysis on heatwaves and their characteristics.

### Caveats

The GHWR data set is constructed based on the CPC gridded global daily temperature record, and its accuracy largely depends on the source data. The CPC gridded temperature data set is constructed using a combination of two large data sets of observation stations around the globe, namely Global Historical Climatology Network version 2 (GCHN)^70,71^ and Climate Anomaly Monitoring System (CAMS)^72^. GCHN and CAMS together provide some 10,978 stations from around the globe (although not consistent through time and space), measured temperatures of which are interpolated to uniform global grids with a spatial resolution of 0.5°×0.5° using an anomaly interpolation method, which is superior to other interpolation techniques in face of sparse station network coverage and temporally missing data^73^. Moreover, temperatures are topographically adjusted using spatiotemporally varying temperature lapse rate estimated from observation-based global reanalysis temperature fields. This data set is comprehensively vetted and contrasted against other products such as Parameter elevation Regression on Independent Slopes Model (PRISM), National Climate Data Center (NCDC) Climate Division Data, Climate Research Unit (CRU) Climatology, Global Climate Data Assimilation System (CDAS) Reanalysis II and ERA40 reanalysis data sets, and rendered a “reasonably good” performance^73^.

Note that GCHN and CAMS stations have a good coverage over the U.S., Europe, Russia and China; and less coverage over central part of Southern America, majority of Africa, and central Australia. Spatiotemporal coverage of observation stations used in CPC data set is displayed in Fig. 2 of Fan and Van den Dool^73^ and can also be accessed through the Climate Prediction Center website. Accuracy of the gridded temperature data set is largely dependent on the spatiotemporal density of observation stations, and hence care is advised when using the GHWR product for areas with very few gauges. However, if lack of accuracy in the source data is manifested in terms of (uniform) bias, it would not impact the percentile-based methods used for identifying heatwaves/warm-spells. Finally, the presented GHWR toolbox allows integrating other data sources for analyzing heatwaves and warm spells.

## Additional information

**How to cite this article**: Raei. E. *et al.* GHWR, a multi-method global heatwave and warm-spell record and toolbox. *Sci. Data*. 5:180206 doi: 10.1038/sdata.2018.206 (2018).

**Publisher’s note**: Springer Nature remains neutral with regard to jurisdictional claims in published maps and institutional affiliations.

## Supplementary Material



## Figures and Tables

**Table 1:** Definitions of heatwave/warm-spells used in global heatwave and warm-spell record.

Method	Temperature type	Temperature threshold (°C)	Temporal persistence (days)	Other	Localized threshold?
Constant Threshold	TMAXTMINTMEAN	35, 36, …, 4525, 26, …, 3530, 31, …, 40	2, 3, 4, 5, 6, 7, and 10	—	None
Average temperature + 5 °C	TMAXTMINTMEAN	Average annual temperature + 5 °C	2, 3, 4, 5, 6, 7 and 10	—	Spatial only
Upper tail percentile	TMAXTMINTMEAN	70, 75, 80, 85, 90, 95^th^ percentile of PDF from:•15 day window centered at calendar day•21 day window centered at calendar day	2, 3, 4, 5, 6, 7 and 10	—	Temporal and spatial
Summer-derived threshold	TMAXTMINTMEAN	70, 75, 80, 85, 90, 95^th^ percentile of PDF from:• June-August • June-September• May-August • May-September	2, 3, 4, 5, 6, 7 and 10	—	Spatial only
EHF	TMEAN	-	3-day average	EHF ≥ 0EHF ≥ 1	Temporal and spatial
SHI	TMEAN	-	3-day average	SHI ≥ 1	Temporal and spatial
